# Macrophage-derived SPARC Attenuates M2-mediated Pro-tumour Phenotypes

**DOI:** 10.7150/jca.39651

**Published:** 2020-03-04

**Authors:** Jianwen Hu, Yongchen Ma, Ju Ma, Shanwen Chen, Xiaoqian Zhang, Shihao Guo, Zhihao Huang, Taohua Yue, Yanpeng Yang, Yingze Ning, Jing Zhu, Pengyuan Wang, Xin Wang, Guowei Chen, Yucun Liu

**Affiliations:** 1 Department of General Surgery, Peking University First Hospital, Beijing, 100034, PR China.; 2 Endoscopy Center, Peking University First Hospital, Beijing, 100034, PR China.

**Keywords:** tumour-associated macrophages, SPARC, gastric cancer, proliferation, migration, apoptosis

## Abstract

Since the theory of seed and soil was put forward, people have increasingly recognized that the tumour microenvironment is an important regulator of tumour progression and therapeutic response. Among them, M2-type macrophages (M2, as the major macrophage subtype in the tumour foci) have important promoting effects on various biological behaviours. Secreted protein acidic and rich in cysteine (SPARC) is an important anti-tumour component in the microenvironment of gastric cancer. This study shows that macrophages are an important source of the SPARC and that SPARC overexpression in M2 can reduce M2-mediated promoting proliferation, migration and anti-apoptotic effects in gastric cancer. Additionally, the AKT/mTOR signalling pathways may participate in the malignant process.

## Introduction

Tumour development is usually regarded as an autonomous process, which involves the genetic transformation of cells. However, since the proposal of the theory of seed and soil, people have increasingly recognized that the tumour microenvironment is an important regulator of tumour progression and therapeutic response 1. Among them, macrophages, which are important components of the tumour microenvironment, have important effects on various biological behaviours, such as tumourigenesis, proliferation, migration, invasion and apoptosis 2-5. The phenotypes and functions of macrophages are regulated by the surrounding environment. Macrophages usually have two different subtypes: (1) classical activation or M1-type macrophages (M1), which can be induced from M0 macrophages (M0) in the initial state by lipopolysaccharide and Th1-related cytokines, such as IFN-γ, M-CSF, and GM-CSF alone or in combination, whose surface markers are HLA-DR and NOS2 5-8; and (2) selective activation or M2-type macrophages (M2), which can be induced by Th2-related cytokines such as IL-4, IL-13, IL-10 and glucocorticoid alone or in combination. M2 can synthesize and secrete TGF-β and CCL14, CCL22 and Arg1, and its surface markers are CD163, CD204, CD206, Stabilin-1, etc. 4, 9, 10. M1 produces pro-inflammatory cytokines that fight against pathogenic infections and inhibit tumours. In contrast, M2 produces various anti-inflammatory factors that not only inhibit the immune system but also promote tumour angiogenesis, proliferation, invasion, metastasis and anti-apoptosis. As the major macrophage subtype in the tumour foci, M2 macrophages are involved in all aspects of tumour development and are also called tumour-associated macrophages (TAMs) 5, 10, 11.

Secreted protein acidic and rich in cysteine (SPARC) is a 32-kDa extracellular non-structural matrix glycoprotein. It was also called osteonectin, BM-40 or 43K protein, which was first isolated and purified in human and foetal bovine bone^12^. It has been proven that SPARC plays roles in tissue remodelling and cell-matrix crosstalk. Recently, emerging evidence has shown that SPARC could also affect the malignant process in various solid tumours, and its function is highly heterogeneous among tumour types 13-15. Tumour cell-derived SPARC was reported to inhibit cell proliferation, invasion and angiogenesis in gastric cancer 14, 16. The expression level of SPARC in gastric cancer cells is low, and the promoter methylation of the SPARC gene is one of the main reasons for its loss of expression. SPARC is negatively correlated with tumour TNM stage and local lymph node metastasis. Previous research results of our research group also found that SPARC is an important tumour suppressor component in gastric cancer 16-18.

In addition, Chen et al. found that SPARC in human gastric cancer tissues was mainly derived from stromal cells 19, 20. By immunohistochemical staining of SPARC in gastric cancer tissues, it was found that SPARC was mainly produced by tumour stroma in gastric cancer, and tumour-associated macrophages, tumour-associated fibroblasts and endothelial cells are the main sources 20-22. However, little research has been conducted to explore the role of macrophage-derived SPARC in tumour progression in gastric cancer. As a tumour suppressor in the microenvironment of gastric cancer, can SPARC regulate the role of M2 in promoting tumours? Based on the studies above, the present study investigated the regulatory effects of endogenous SPARC on M2-mediated tumour growth promotion and the potential of macrophage-derived SPARC as a biomarker for the prognosis of gastric cancer and as a potential therapeutic target.

## Materials and Methods

### Clinical material

Seventy-six patients with primary gastric cancer underwent surgery between 2008 and 2013 at Peking University First Hospital, Beijing, China, and were enrolled in this study in which they received tailored follow-up for 5 years (see Table 1). Sixty-seven patients underwent radical resection of gastric cancer, and nine received palliative resection. We excluded patients who underwent neoadjuvant treatment, such as radiotherapy or chemotherapy, before surgery from this study. The pathological diagnosis was confirmed by doctors from the Department of Pathology, Peking University First Hospital, and the classifications of gastric cancer were made based on the TNM Staging Classification for Carcinoma of the Stomach formulated by the American Joint Committee on Cancer (8th, 2018). This study was approved by the Peking University First Hospital Biomedical Research Ethics Committee (No. 2017-37**)**. All patients involved in the project signed informed consent.

### Immunohistochemistry

Paraffin-embedded samples from 76 gastric cancer patients were immunostained with a rabbit anti-human SPARC antibody (1:600; Cell Signaling Technology, Massachusetts, USA). Next, horseradish peroxidase (HRP)-conjugated goat anti-rabbit IgG (ZSGB-BIO, Beijing, China) was incubated for 30 minutes. The DAB staining system was used to indicate SPARC expression. Macrophage-derived SPARC was evaluated by two independent studies, mainly according to the staining intensity and the proportion of positive macrophages. In brief, the intensity scoring was as follows: 0, complete absence of staining or staining in ≤ 5% of cells of the same type; 1, staining in 6% to 25% of cells; 2, staining in 26% to 50% of cells; and 3, staining in > 50% of cells. The sum of these two scores represented the expression level of SPARC: scores of < 4 were categorized as low expression; and scores of ≥ 4 were considered high expression 23. CD163 (1:1000; Abcam, Cambridge, England) and Stabilin-1 (1:250; Santa Cruz Biotechnology, California, USA) antibodies were used to localize macrophages in gastric cancer tissues.

### Cell line culture

The gastric cancer cell lines HGC-7901 and BGC-823 were purchased from the Cancer Institute of the Chinese Academy of Medical Science. The monocyte cell line THP-1, human normal gastric mucosal epithelial cell line GES-1 and the gastric cancer cell line MKN-45 were purchased from The Global Bioresource Center (ATCC). HGC-7901, BGC-823 and GES-1 were cultured in Dulbecco's modified Eagle's medium (DMEM, Thermo Fisher Scientific, Waltham, MA, USA), while Roswell Park Memorial Institute 1640 (RPMI 1640, Thermo Fisher Scientific, Waltham, MA, USA) was used to culture THP-1 and MKN-45 in the optimal conditions of 37℃ with 5% CO_2_. Both types of medium were supplemented with 10% foetal bovine serum (FBS, Thermo Fisher Scientific, Waltham, MA, USA) and 1% penicillin-streptomycin solution (Thermo Fisher Scientific, Waltham, MA, USA).

### Differentiation of THP-1 cells, acquisition of conditioned medium (CM) and co-culture system

To generate THP-1-derived M2 macrophages (M2), THP-1 cells were treated with 150 nM phorbol 12-myristate 13-acetate (PMA, Sigma-Aldrich, MO, USA) for 24 hours to become activated macrophages (M0) and then were cultured with new media containing 20 ng/ml IL-4 and 20 ng/ml IL-13 for another 48 hours to become M2. Real-time Polymerase Chain Reaction was employed to detect the mRNA expression level of CD163, stabilin-1, IL-10, and Arg1 in M2 to verify the successful induction 4, 8, 24, 25. After the induction by IL-4 and IL-13, differentiated M2 cells were washed in fresh PBS and cultured in RPMI 1640 medium. The CM was obtained after 48 hours of culture and was collected and stored at -80℃ for subsequent use^25^. Tumour cells were cultured in conditioned medium for the transwell migration test, Cell Counting Kit-8 and cell colony formation assays. Lentiviral-infected SPARC-regulated THP-1 cells were also induced to the corresponding SPARC-regulated M2 (M2-OE and M2-VEC) by the same induction method.

### Real-time PCR

Total RNA was isolated using the TRIzol method (Thermo Fisher Scientific, Waltham, MA, USA). RNAs were transcribed into cDNAs using a High-Capacity RNA-to-cDNA kit (Thermo Fisher Scientific, Waltham, MA, USA). Real-time PCR was performed using the 7900HT Fast Real-Time PCR system (Applied Biosystems, Darmstadt, Germany). Expression levels were normalized using GAPDH. The relative expression was calculated by the 2^(-ΔΔCt)^ method. The primer sequences are available below.

### Cell Counting Kit-8 (CCK-8)

BGC-823, SGC-7901, MKN-45 and GES-1 cells were treated in M2 conditioned medium (CM) at a gradient concentration (10% to 80%, with an interval of 10%), and the rest was supplemented with FBS-free medium in 96-well plates. A total of 8000 tumour cells were added to each well. After 48 hours, the cell proliferation status was examined using the Cell Counting Kit-8 (Bimake, Shanghai, China). CCK-8 reagent was applied and incubated for 40 minutes. The absorbance at 450 nm was measured.

### Cell colony formation assay

Tumour cells and GES-1 were cultured in 12-well plates at 300 cells per well. They were incubated with a certain proportion of CM for 10 days to detect cell colony formation ability. The ratio of conditioned medium to normal serum-free medium was 3/4. cells were fixed with 10% formaldehyde for 20 minutes and stained with 0.1% crystal violet for 30 minutes at room temperature. The staining solution was then discarded carefully, and each well was washed with water. The plates were then inverted on absorbent paper to dry. Finally, the cells were visualized in five fields under a microscope (TE2000-S, Nikon, Tokyo, Japan). The number of cells was counted using ImageJ (×64) (National Institutes of Health, Maryland, USA). The results are expressed as the average number of cells in every visual field.

### Transwell migrating assay

A total of 1×10^5^ BGC-823, SGC-7901 or GES-1 cells were plated on 8-μm pore polycarbonate inserts (Corning, New York, USA), and 0.8 ml of CM was added to the lower chamber. In addition, 0.2 ml of serum-free medium was added to maintain a basic acid-base environment suitable for cell survival. After 24 hours, cells penetrating the lower surface of the transwell chamber were fixed with methanol and stained with crystal violet. Cells were counted in five randomly selected fields for each sample under microscopy and photographed. Three duplicate holes for each experimental group were set up.

### Establishment of the tumour xenograft models and *in vivo* imaging experiment

Four- to six-week-old nude mice were obtained from the experiment. The experimental animal procedures were approved by the Experimental Animal Welfare Ethics Committee of Peking University First Hospital (201738). BGC-823 gastric cancer cells (1×10^6^) infected by lentivirus with the luciferase gene were mixed with M2 (1:1) and then were subcutaneously injected into the nude mice at the axilla region. After visually estimating the difference between the experimental group and the control group, *in vivo* imaging experiments were performed. Mice were injected intraperitoneally with D-Luciferin firefly, sodium salt monohydrate (Promega, Wisconsin, USA), and photographs were taken with Spectrum Living Image 4.0 every three days before reaching 1.5 cm. Finally, the mice were sacrificed.

### Analysis of apoptosis

For the apoptosis assay, BGC-823, SGC-7901 and MKN-45 cells were incubated in SPARC overexpressed-M2 conditioned medium in the presence or absence of 1 μg/mL 5-fluorouracil for 72 hours 18. GES-1 cells were incubated in SPARC overexpressed-M2 conditioned medium in the presence or absence of 30 μg/mL 5-fluorouracil for 48 hours. Then, the cells were trypsinized and washed with serum-containing medium. Cells were then centrifuged for 3 minutes at 1500 rpm, and the supernatant was discarded. Cell apoptosis was assessed with the Annexin V-FITC/PI Apoptosis Analysis Kit (Becton, Dickinson, New Jersey, USA). After harvesting, the cells were washed twice with cold PBS and resuspended in 100 μL of 1×Annexin binding buffer. Then, 5 μL of Annexin V-FITC and 5μL of Propidium Iodide (PI) solution were added to the cell suspension and incubated at 37°C for 15 minutes. The stained cells were analysed with a FACS system (FACSCalibur, Becton, Dickinson, New Jersey, USA).

### Lentivirus packaging and infections

SPARC cDNA (NM_003118) was inserted into the plasmid pLenti-EF1a-MCScarlet-P2A-Puro (Mailgene Biosciences, Beijing, China) to make a recombinant plasmid for the overexpression of SPARC in THP-1 cells. HEK293T cells were transfected with the plasmid in association with the lentivirus backbone plasmids psPAX-2 and pMD2.G. After 48 hours, the culture medium was harvested, and lentivirus in the medium was purified. The optimal MOI for the infection of THP-1 cells was determined before use. The parent plasmid pLenti-EF1a-MCScarlet-P2A-Puro underwent the same procedure to obtain a lentivirus as a negative control. THP-1 cells were infected with the lentivirus. After infection for 72 hours, approximately 10% of the cells presented red fluorescence. Puromycin (1μg/ml) was then added to the medium until 30% of the cells displayed red fluorescence, and these cells were then isolated by flow cytometry and cultured for expansion of the positive cells. THP-1 cells stably overexpressing SPARC and infected with the negative control lentivirus were designated THP-OE and THP-VEC, respectively.

### Western blot analysis

The expression levels of proteins in cancer cells were examined as follows. Total cellular proteins were prepared from cell lysates with lysis buffer. For AKT-mTOR signalling pathway protein detection, cells were treated with OE/VEC CM for 30 minutes before protein extraction. After the protein concentration of each sample was adjusted, SDS-polyacrylamide gel electrophoresis was performed to separate proteins. Subsequently, the protein bands were transferred to a polyvinylidene fluoride (PVDF) membrane. The specific primary antibodies were used as follows: p-PTEN (1:1000, Cell Signaling Technology, Massachusetts, USA), p-AKT (1:1000, Cell Signaling Technology, Massachusetts, USA), AKT (1:1000, Cell Signaling Technology, Massachusetts, USA), p-mTOR (1:1000, Cell Signaling Technology, Massachusetts, USA), mTOR (1:1000, Cell Signaling Technology, Massachusetts, USA), p-70S6K (1:1000, Cell Signaling Technology, Massachusetts, USA), and GAPDH (1:1000, Cell Signaling Technology, Massachusetts, USA) at 4°C overnight. GAPDH served as the internal control. The levels of target proteins were detected using the ECL detection system (Merck, Darmstadt, Germany) and the Syngene GeneGenius gel imaging system (Syngene, Cambridge, UK). The relative changes in protein expression were analysed using ImageJ software.

### Statistical analyses

The correlations between SPARC expression and clinicopathology were evaluated by χ2 test (for dichotomous data) or the Kruskal-Wallis test (for ranked ordinal data). Kaplan-Meier analysis was applied to calculate the survival duration, and the significance between groups was analysed using the Breslow (Generalized Wilcoxon) test. Cox regression analysis was employed to compute multivariate hazard ratios (HRs) for the parameters. A t-test was used for the two groups in the transwell assay, cell colony formation assay, CCK-8 and the apoptosis assay. The results of all tests noted above were analysed using SPSS 20.0 software, and all tests were two-tailed. P values less than 0.05 were considered statistically significant.

## Results

### Macrophages and fibroblasts are the main sources of SPARC in gastric cancer foci, and the expression of SPARC in macrophages is negatively correlated with the prognosis

**Figure 1:**
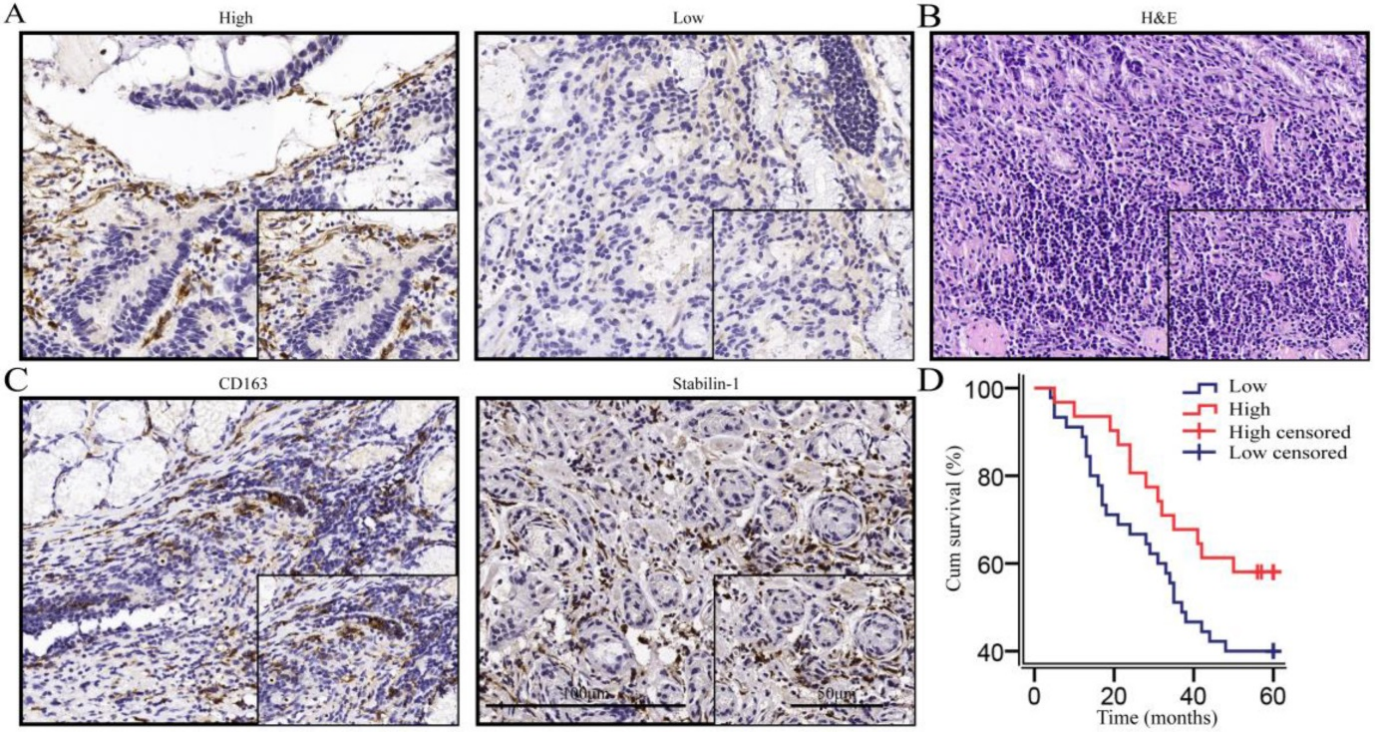
**The expression of SPARC was high in macrophages, and high SPARC expression was associated with a favourable prognosis in the trend. A.** Immunohistochemical staining indicated that macrophages and fibroblasts were the main sources of SPARC; **B.** HE staining of gastric cancer tissues; **C.** CD163 and Stabilin-1 localized macrophages (mainly M2) in gastric cancer tissues;** D.** The survival analysis showed that the high expression of SPARC in macrophages was beneficial to the prognosis in the trend (*P*=0.078).

We performed immunohistochemical staining on 76 gastric cancer tissues according to the calculated minimum sample size and reached the cell structure level for staining intensity count and survival analysis. Immunohistochemical morphology showed that macrophages were the important sources of SPARC (Fig. 1A). Haematoxylin-eosin (HE) staining was used to indicate gastric cancer tissue (Fig. 1B). CD163 and Stabilin-1, as M2-associated markers, were used to localize macrophages in gastric cancer tissues (Fig. 1C). Seventy-six patients with gastric cancer were enrolled for a 5-year-long follow-up. The survival curves showed that the overall survival rate of macrophage-derived SPARC patients with low expression of gastric cancer was lower than that of patients with high expression of SPARC, but the difference was not statistically significant (χ2=3.106, *P* = 0.078, Fig. 1D). A correlation test found that macrophage SPARC expression was negatively correlated with lymph node metastasis (*P* =0.017, Table 1). Univariate and multivariate COX regression analyses were performed on multiple indicators affecting the prognosis of patients with gastric cancer (Table 2). Univariate analysis showed that OS was significantly correlated with tumour location, lymph node metastasis and distant metastasis. Multivariable Cox regression analysis demonstrated that lymph node metastasis (HR =2.383, *P* =0.022, Table 2) and distant metastasis (HR =6.115,* P* <0.001, Table 2) are independent predictive factors for OS in gastric cancer.

### Construction of the M2 and SPARC overexpression M2 model

**Figure 2:**
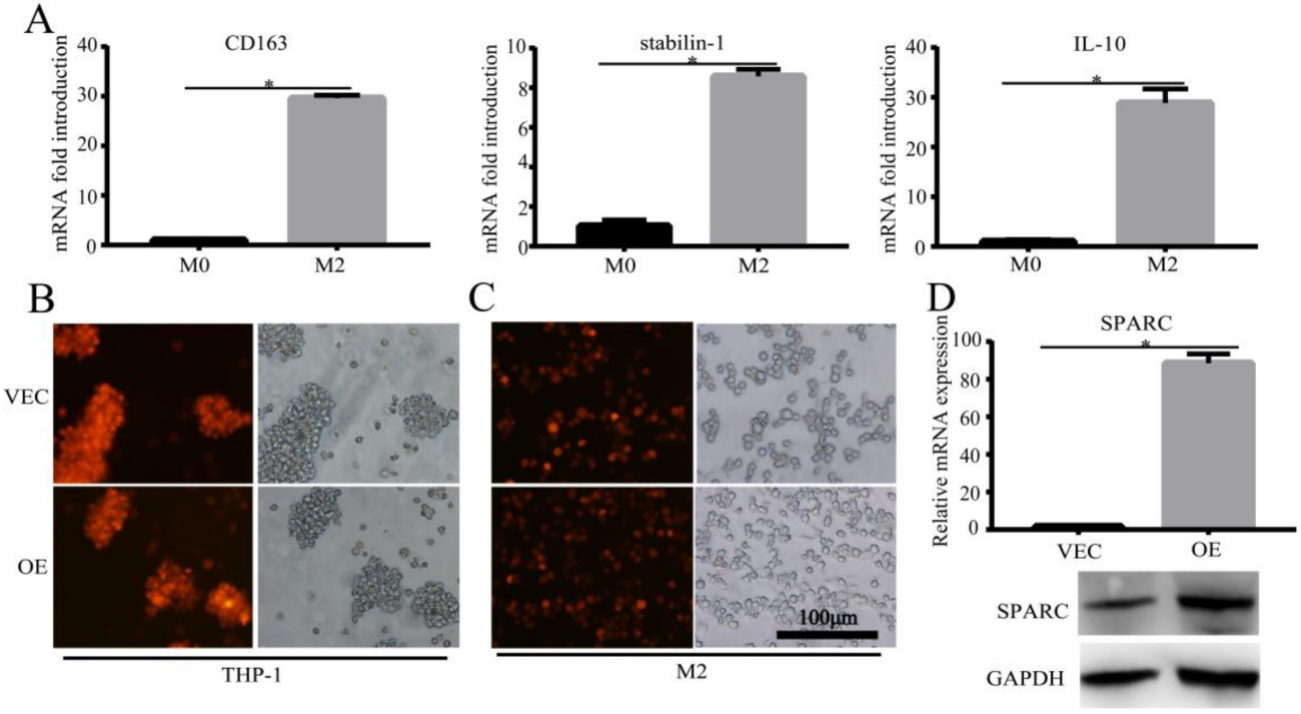
** Construction of the M2 and SPARC overexpression M2 model. A**. M2-related marker expression (CD163, Stabilin-1 and IL-10) was higher than M0, which indicated that the M2 model was successfully constructed. **B, C.** The SPARC gene sequence was inserted into THP-1 cells by lentivirus infection, and THP-1 cells were induced into M2 by PMA and IL-4/IL-13 sequentially. The mScarlet fluorescence label implied successful transfection. **D.** RT-PCR and western blot showed that compared with M0, SPARC was overexpressed in M2 at the mRNA and protein levels, respectively. Statistical analysis was carried out with a two-sample *t*-test. The results are shown as the means (±s.d.). *P < 0.05, **P < 0.01.

M2 is the major macrophage subtype in the tumour area. In order to verify the effect of SPARC overexpression in M2 on the macrophage-mediated malignant tumour phenotype, we first needed to construct the M2 cell model and the regulated SPARC expression M2 cell model. Then, the corresponding conditioned medium and M2 cells were co-cultured with gastric cancer cell lines for *in vitro* and *in vivo* experiments. We sequentially induced the mononuclear cell line THP-1 into M2-type macrophages using PMA and interleukin-4 and -13 (IL-4/13). CD163, Stabilin-1 and IL-10 mRNA (M2 markers) expression was significantly higher in M2 than in M0 (*P*<0.05) (Fig. 2A), which showed the success of the M2 model construction. After lentivirus infection and induction by cytokines, RT-PCR and western blot showed that SPARC was overexpressed in M2 compared with M0 (Fig. 2D), which illustrated that the SPARC overexpression M2 model was successfully established.

### SPARC overexpression in M2 reduced M2-mediated proliferation of gastric cancer

**Figure 3:**
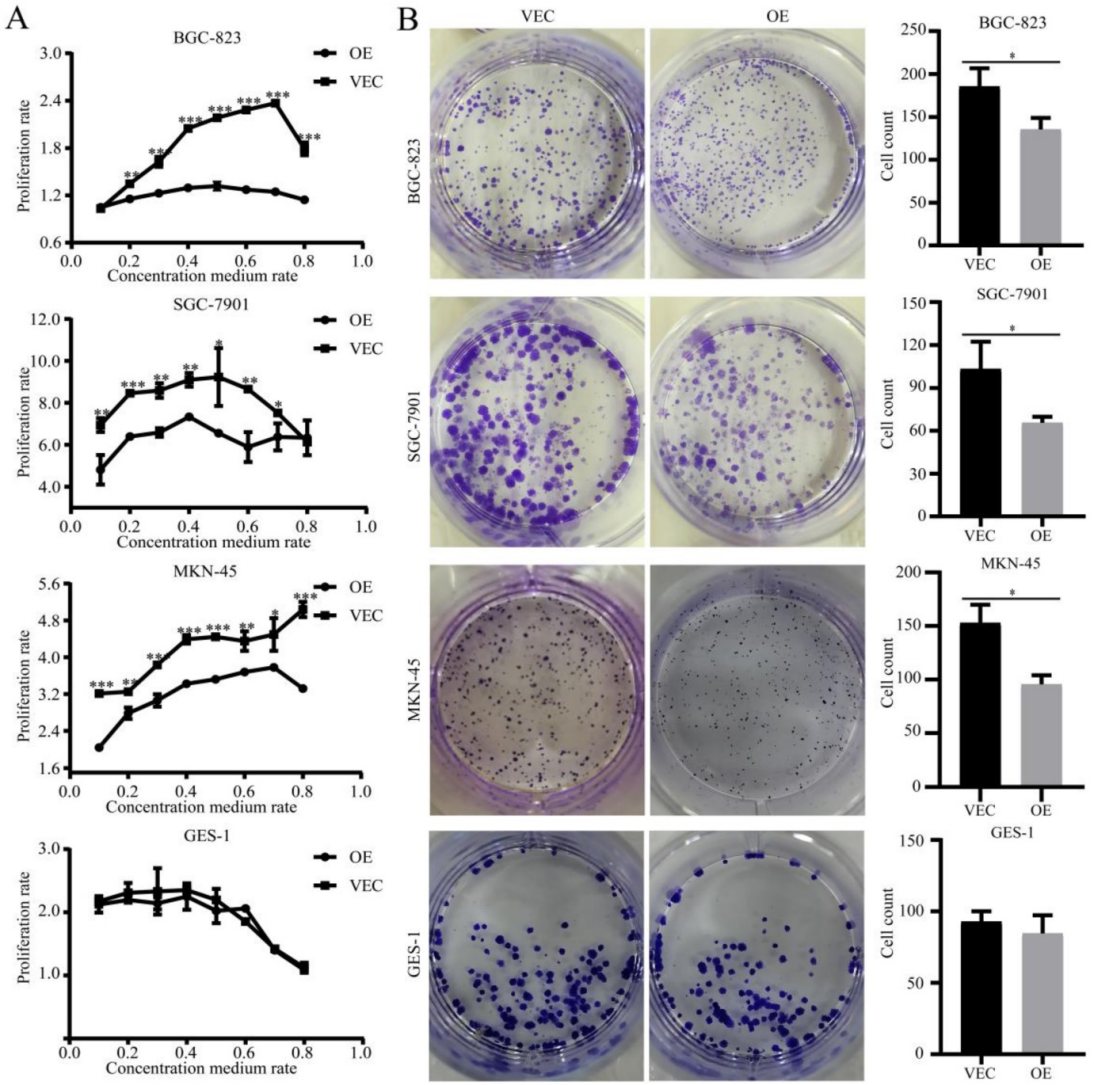
** Effects of SPARC overexpression in M2 on M2-mediated proliferation of gastric cancer.** The cell proliferation ability was assayed by the CCK-8 method **(A)** and cell colony formation assay** (B)**. SPARC overexpression in M2 reduced M2-mediated proliferation of gastric cancer, but has no effect on GES-1 proliferation. Experiments were repeated three times, and the luminous intensity was measured three times. * *P* < 0.05, ** *P* < 0.01, *** *P* < 0.001.

To determine the effects of SPARC overexpression on M2-mediated proliferation of gastric cancer, we performed a CCK-8 assay and a colony formation assay. CCK-8 showed that the growth of gastric cancer cells, which were co-cultured with SPARC overexpression CM, slowed compared with that of the control group *(P*<0.05) (Fig. 3A). Similarly, the former had a significantly reduced ability to form clones compared to that of the control group (*P*<0.05) (Fig. 3B). The two assays illustrated that SPARC overexpression in M2 can reduce M2-mediated proliferation of gastric cancer. No significant differences were observed on normal gastric mucosal GES-1 cells in proliferation experiments.

### Detection of the migration ability and phenotype-associated protein expression of gastric cancer cells *in vitro*

**Figure 4:**
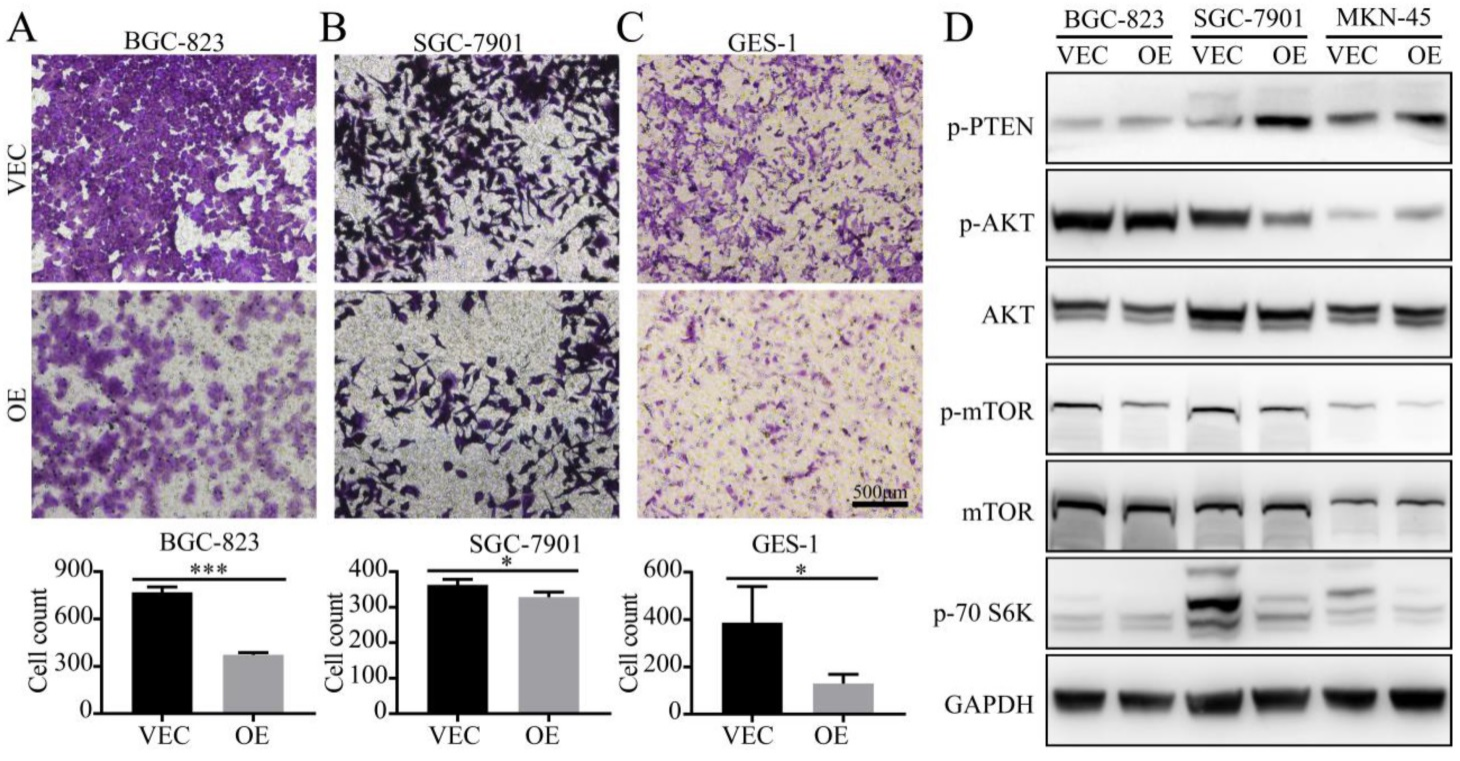
** Detection of the migration ability of gastric cancer cells *in vitro*. A, B, C.** BGC-823, SGC-7901 and GES-1 cells were co-cultured in the CM from M2 (OE CM or VEC CM). The ratio of normal medium to conditioned medium was 3:1. The cells that migrated through the transwell were stained with crystal violet and counted using ImageJ. The results are shown as the means (±s.d.). D**.** OE CM-treated gastric cancer cells expressed low levels of p-mTOR, p-AKT, and p-70S6K.

To demonstrate the effects of SPARC on the M2-mediated proliferation of gastric cancer, we performed a transwell migration assay. When BGC-823 and SGC-7901 cells were cultured in SPARC overexpressing M2 conditioned medium, the number of tumour cells that could pass through the upper chamber was significantly reduced compared with that of the control group (Fig. 4). These results indicate that SPARC overexpression in M2 can attenuate M2-mediated gastric cancer migration. The same results were observed in GES-1 transwell migration assay (Fig.4).

To investigate the protein changes in the expression levels of gastric cancer after treatment with OE/VEC CM, M2-OE and M2-VEC cells were analyzed via western blot. We identified that OE CM-treated gastric cancer cells had low expression of p-mTOR, p-AKT, and p-70S6K, which illustrated that OE CM may inhibit the AKT/mTOR signalling pathway and attenuate M2-mediated malignant phenotypes.

### SPARC overexpression in M2 reduces M2-mediated anti-apoptosis ability

**Figure 5:**
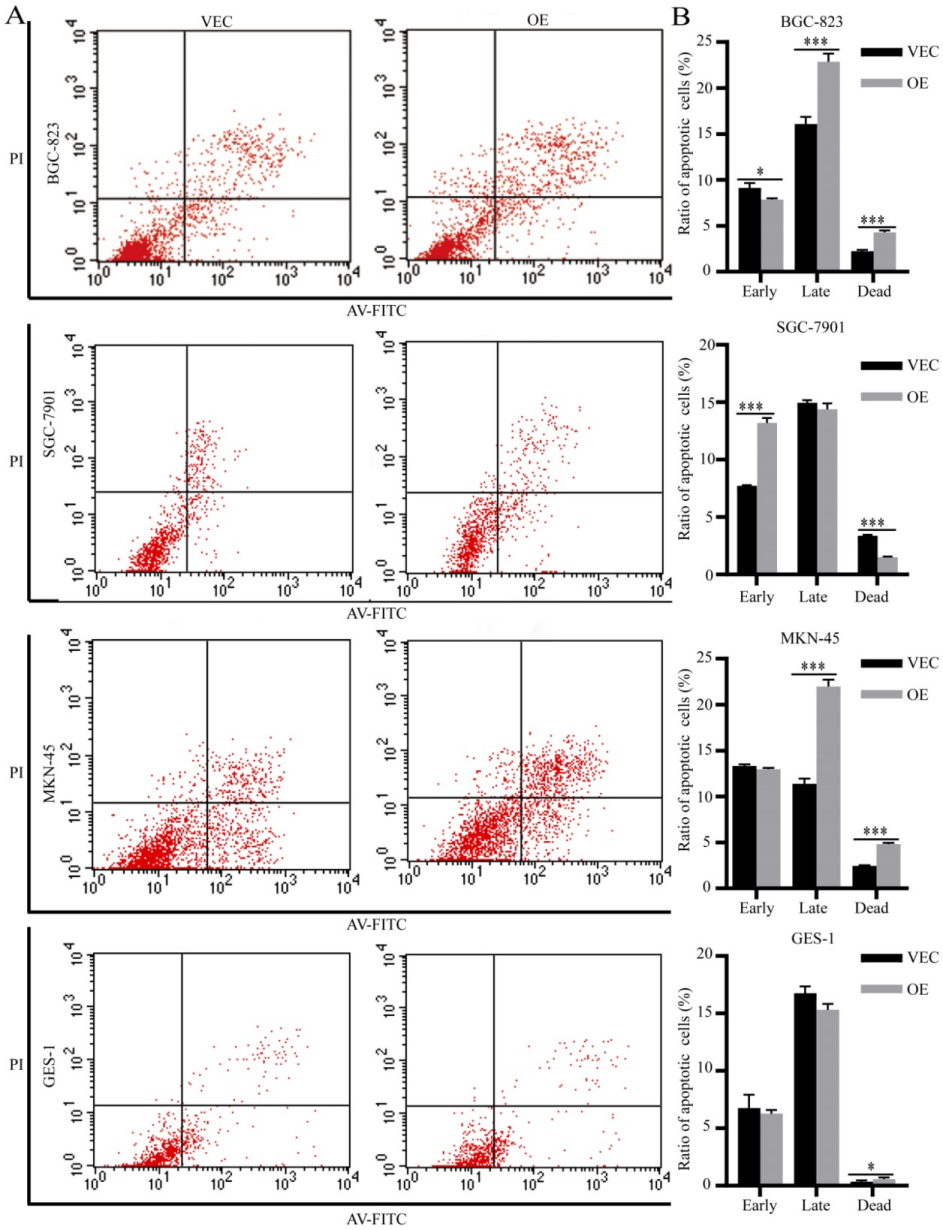
** Effects of SPARC overexpression on M2-mediated anti-apoptosis ability.** After treated with 5-fluorouracil at IC50 (1 µg/mL for BGC-823, SGC-7901, MKN-45 and 30 µg/mL for GES-1) for 48 hours, more apoptotic cells were observed by flow apoptosis detection in all four SPARC overexpression groups.

We performed flow apoptosis detection to evaluate the effects of SPARC on M2-mediated anti-apoptosis ability. After treatment with 5-fluorouracil at the half maximal inhibitory concentration (IC50) for 48 hours, the ratio of apoptotic cells was measured by flow cytometry. Compared with the control group, the BGC-823 and MKN-45 gastric cancer cells treated with SPARC-treated M2 medium showed increased late apoptosis and dead levels (Fig. 5A, C). The SGC-7901 gastric cancer cells treated with SPARC-treated M2 medium showed an increased apoptosis level at the early stages (Fig. 5B). The normal gastric mucosal cells GES-1 incubated with SPARC-treated M2 medium showed an increased dead level (Fig. 5D). The results indicated that SPARC overexpression attenuates M2-mediated anti-apoptosis ability.

### SPARC overexpression in M2 reduced the growth rate of tumour xenografts in nude mice

**Figure 6:**
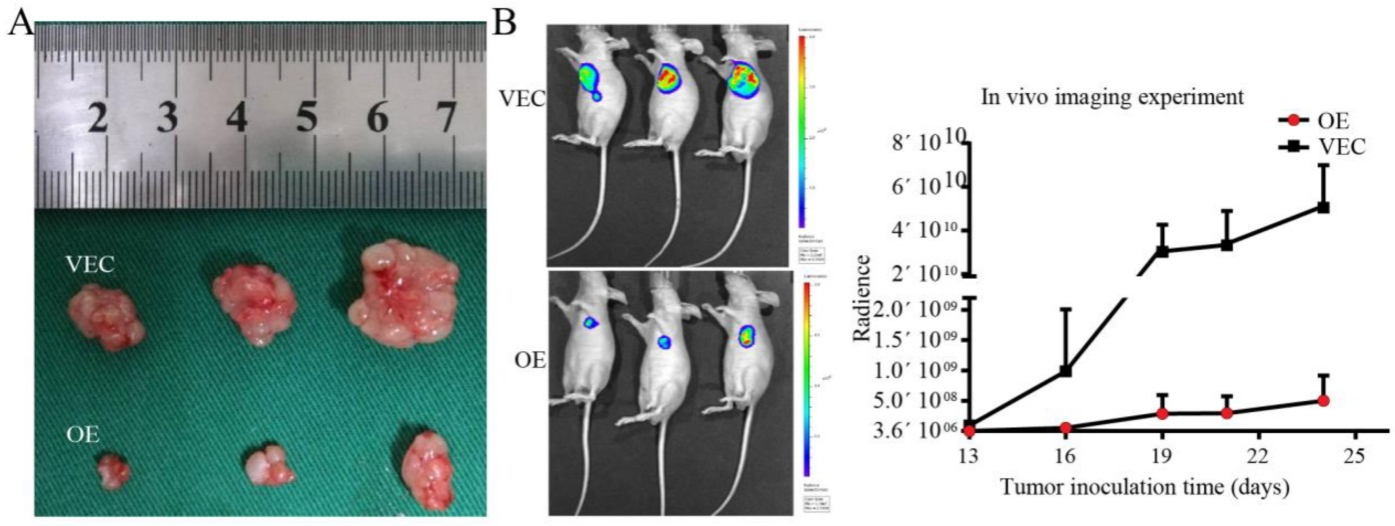
** Establishment of tumour xenografts. A.** The growth rate of SPARC-overexpressing M2 and BGC-823 simultaneously injected subcutaneously into nude mice was lower than that of the control group. **B.** *In vivo* imaging showed the size of the transplanted tumour.

The results of *in vivo* imaging experiments showed that SPARC-overexpressing M2 and BGC-823 gastric cancer cells were simultaneously injected subcutaneously into nude mice, and their growth rate was much lower than that of the control group. *In vivo* experiments showed that SPARC overexpression can significantly reduce the role of M2 in promoting tumour growth (*P*<0.05, Fig. 6).

## Discussion

The tumour microenvironment includes tumour- associated cellular components and non-cellular matrix components, which are composed of an inseparable and interactive system for tumorigenesis. Tumour-associated macrophages, as one of the most important cells in the microenvironment, have various effects on tumour progression and poor prognosis 11, 26. Many studies have shown that SPARC is an important tumour suppressor in the gastric cancer microenvironment. SPARC suppresses angiogenesis by downregulating the expression of VEGF and MMP-7 in gastric cancer 17. Additionally, SPARC regulates angiogenesis by sequestering VEGF, thus restricting the activation of VEGF receptor and ERK1/2, consequently limiting the proliferation of cancer cells 27. On the other hand, some studies and our previous work have found that SPARC was mainly derived from the stroma, such as macrophages, and the immunohistochemical staining in the present study also showed the same SPARC expression distribution 22. Although previous studies have focused on the effects of tumor-derived SPARC on biological behaviours such as tumour proliferation and migration, the behaviours of SPARC from macrophages remains poorly understood, because the roles of SPARC from different cell origins are highly heterogeneous. Subsequently, this study further explored the role of SPARC derived from macrophages in M2-mediated cancer malignant phenotypes.

In the present study, we evaluated the expression level of SPARC in 76 gastric cancer samples. Cell morphology identification and SPARC expression counting in macrophage were performed by pathologists, which accurately illustrated the prognostic value of SPARC derived from the macrophage rather than the fibroblast and the endothelial cells. Macrophage SPARC expression was negatively correlated with T staging, TNM staging, number of lymph node metastases, tumour location, and lymph node metastasis. Therefore, we formed a preliminary hypothesis: SPARC as a protective component can reduce M2-mediated gastric malignant phenotypes. High expression of macrophage-derived SPARC favours prognosis. Because we examined only 76 patients according to the minimum sample size estimation method, the results may be random due to the heterogeneity of gastric cancer. The results need to be confirmed in a large study. A correlation test found that macrophage SPARC expression was negatively correlated with lymph node metastasis, which suggested that macrophage-derived SPARC may be a protective factor.

Many studies have demonstrated that M2 can promote malignant behaviours such as tumour proliferation, migration, and anti-apoptosis 28. In order to verify the roles of SPARC in these behaviours, tumour cells were co-cultured with SPARC-regulated M2 or cultured in conditioned medium. The CCK-8 assay, colony formation assay and tumour xenograft experiment demonstrated that SPARC could reduce the tumour-promoting effects of M2. The *in vivo* imaging experiment showed that the proliferation rate of the transplanted tumours in the experimental group was much lower than that of the control group. The three assays illustrate that SPARC overexpression in M2 attenuated M2-mediated proliferation of gastric cancer. Overexpression of SPARC had no effect on the proliferation of normal gastric mucosal cell GES-1. This provides a guarantee for future SPARC-targeted treatment on gastric cancer, because it only attenuates the proliferation of gastric cancer cells, but does not affect the proliferation of normal gastric mucosa.

There has been disagreement regarding the migration functions of endogenous SPARC. Although Sangaletti et al. showed that macrophage-derived SPARC induces cancer cell migration and enhances their migration to other extracellular matrix proteins, at least through alpha (v) beta (5) integrin 21, the same effect was not observed in the present study. Transwell assays illustrated that macrophage-derived SPARC can decrease the pro-migration function of M2. This may be due to the former study using SPARC knockout mice for animal experiments, which was heterogeneous with human cells.

Many studies have shown that activation of the AKT/mTOR pathway is an important mechanism for tumour proliferation, migration, and anti-apoptotic ability 29, 30. There are also studies that show that macrophages play a role in tumour growth by activating tumour AKT/mTOR pathways. TAMs promote epithelial mesenchymal transition via activating the PI3K/AKT/mTOR signalling pathway in endometrial cancer 31. Infiltrating macrophages increase RCC epithelial mesenchymal transition (EMT) and stem cell-like populations via AKT and mTOR signalling 32. Therefore, we hypothesized that the overexpressed SPARC M2 conditioned medium may reduce the activation of the AKT/ mTOR pathway in tumour cells, thereby attenuating the M2-mediated malignant tumour phenotype. To further explore the molecular mechanisms of SPARC derived from macrophages in regulating M2-mediated pro-phenotypes, western blotting was performed to detect AKT/mTOR pathway activation. Western blot OE CM-treated gastric cancer cells expressed low levels of p-mTOR, p-AKT, and p-70S6K, which illustrated that OE CM may inhibit the AKT/mTOR signalling pathway and attenuate M2-mediated malignant phenotypes.

Chemotherapy resistance and anti-apoptosis are major obstacles to adjuvant treatment of malignant tumours, which seriously affects the long-term survival of patients with gastric cancer. The 5-fluorouracil-induced apoptosis assay showed that the ratio of BGC-823 and MKN-45 cultured in SPARC-overexpressing M2 conditioned medium was significantly higher than that of the control group. However, the early apoptosis of SGC-7901 cells increased, which may be due to cell line heterogeneity. The normal gastric mucosal cell GES-1 incubated with SPARC-treated M2 medium also showed an increased dead level. The results illustrated that M2 SPARC overexpression can reduce M2-mediated resistance and apoptosis resistance of tumour cells.

The M2 model which is widely recognized induced from THP-1 and regulated by SPARC expression was applied in our study. This method of the M2 model induced from THP-1 has been used in many studies to construct macrophage models, and this study truly illustrated that M2-mediated physiological status changes in the tumour process after SPARC overexpression to some extent, but the direct target of SPARC was not found. In the follow-up study, the direct target of SPARC protein in gastric cancer will be found, and the in-depth mechanism of SPARC protein affecting gastric cancer will be explained, which will provide theoretical evidence for clinical treatment and targeted gene therapy. This study demonstrates that the expression of SPARC in M2 can inhibit the progression of gastric cancer, and the initial findings may be achieved by regulating the AKT/mTOR pathway; this study is important for elucidating the regulation of tumour microenvironment-associated macrophages on tumour cells. It provides new evidence for revealing the tumour microenvironment-promoting mechanism and provides scientific support for developing better antitumour immunotherapy methods and searching for potential targets.

In conclusion, M2-derived SPARC is an important protective factor in tumour region inhibition of tumour progression. Endogenous SPARC plays a significant role in reducing M2-mediated proliferation, migration and 5-fluorouracil resistance of gastric cancer. SPARC may be a potential marker for clinical diagnosis.

## Figures and Tables

**Table 1:** The clinicopathological characteristics of 76 patients with gastric cancer

	Number (N)	Macrophage SPARC expression		P-value
		High expression	Low expression	
**Sex**				
Male	59	30 (50.8%)	29 (49.2%)	0.118
Female	17	5 (29.4%)	12 (70.6%)	
**Age**				
>50 years	63	29 (46.0%)	34 (54.0%)	0.994
≤50 years	13	6 (46.1%)	7 (53.8%)	
**Tumour Location**				
Localized	69	48 (69.6%)	21 (30.4%)	0.078
Diffused	7	2 (28.6%)	5 (71.4%)	
**Tumour Size (cm)**				
<5	26	14 (53.8%)	12 (46.2%)	0.326
≥5	50	21	29	
**Histological type**				
Adenocarcinoma	49	23 (47%)	26 (53%)	0.835
Other type	27	12 (44.4%)	15 (55.6%)	
**Grade**				
Well and Moderately differentiated	14	4 (28.6%)	10 (71.4%)	0.303
Poorly differentiated	62	27 (43.5%)	35(56.5%)	
**Degree of invasion**				
T1	3	3 (100%)	0	0.5713
T2	12	5 (41.7%)	7 (58.3%)	
T3	34	15(44.1%)	19 (55.9%)	
T4	27	12 (44.4%)	15 (55.6%)	
**Lymph node metastasis**				
0-6	43	25 (58.1%)	18 (41.9%)	0.017
7 or more	33	10 (30.3%)	23 (66.7%)	
**Remote metastasis**				0.241
No	67	33 (49.3%)	34 (50.7%)	
Yes	9	2 (22.2%)	7 (77.8%)	
**TNM classification**				
Ⅰ	7	5 (71.4%)	2 (28.6%)	0.078
Ⅱ	12	6 (50%)	6 (50%)	
Ⅲ	48	22 (45.8%)	26 (54.2%)	
Ⅳ	9	2 (22.2%)	7 (77.8%)	
**Cancer embolus**				
Yes	45	24 (53.3%)	21 (46.7%)	0.125
No	31	11 (35.5%)	20 (65.5%)	

Categorical variables are expressed as percentages and compared using Pearson's chi-square or Fisher's exact test as appropriate.

**Table A:** 

**GAPDH**	forward	5'-CTGGGCTACACTGAGCACC-3'
	reverse	5'-AAGTGGTCGTTGAGGGCAATG-3'
**Stabilin-1**	forward	5'-CCGGGAAATCCTTACCACAGC-3'
	reverse	5'-ACCTTCGTGTTTGTTGGGTCC-3'
**CD163**	forward	5'-CACCAGTTCTCTTGGAGGAACA-3'
	reverse	5'-TTTCACTTCCACTCTCCCGC-3'
**IL-10**	forward	5'-GTGATGCCCCAAGCTGAGA-3'
	reverse	5'-CACGGCCTTGCTCTTGTTTT-3'

**Table 2:** Univariate and multivariate COX regression analysis of prognosis in patients with gastric cancer.

	Univariate analysis					Multivariate analysis (N=76)			
		95% confidence interval					95% confidence interval		
	Hazard ratio	Lower	Upper	P		Hazard ratio	Lower	Upper	P
**Tumour Location**									
Diffused	2.835	1.184	6.791	0.019		2.226	0.837	5.676	0.094
**Histological type**									
Adenocarcinoma	0.768	0.408	1.447	0.414		0.578	0.277	1.209	0.145
**Grade**									
Poorly differentiated	0.980	0.451	2.128	0.959		0.643	0.260	1.590	0.339
**Degree of invasion**									
T3-T4	1.806	0.707	4.613	0.217		0.935	0.325	2.693	0.901
**Lymph node metastasis**									
7 or more	2.648	1.401	5.008	0.003		2.383	1.131	5.022	0.022
**Remote Metastasis**									
Yes	3.348	1.527	7.342	0.003		6.115	2.956	12.647	0.000
**Cancer embolus**									
Yes	1.226	0.657	2.287	0.522		0.804	0.406	1.591	0.531
**Macrophage SPARC expression**									
High	0.574	0.296	1.113	0.101		0.665	0.323	1.370	0.269

## Abbreviations

TAM: Tumour-associated macrophage
M0: Activated macrophage
M1: M1-type tumour-associated macrophage
M2: M2-type tumour-associated macrophage
HRP: Horseradish peroxidase
CM: Conditional medium
OE CM: Conditional medium for SPARC overexpression M2
VEC CM: Conditional medium for SPARC overexpression M2 in no-load control
THP OE: THP-1 SPARC overexpressed
THP VEC: THP-1 SPARC overexpressed in no-load control
DMEM: Dulbecco's modified Eagle's medium
RPMI 1640: Roswell Park Memorial Institute 1640
ATCC: American Type Culture Collection
FBS: Foetal bovine serum
PBS: Phosphate-buffered saline
SPARC: Secreted protein acidic and rich in cysteine
H&E: haematoxylin and eosin
HR: hazard ratio
